# Silver-Treated Sutures for the Prevention of Biofilm-Associated Surgical Site Infections

**DOI:** 10.3390/antibiotics14010049

**Published:** 2025-01-08

**Authors:** Federica Paladini, Angelica Panico, Annalia Masi, Francesca Russo, Alessandro Sannino, Mauro Pollini

**Affiliations:** 1 Department of Experimental Medicine, University of Salento, Via Monteroni, 73100 Lecce, Italy; federica.paladini@unisalento.it (F.P.); alessandro.sannino@unisalento.it (A.S.); 2 Caresilk S.r.l.s., c/o Dhitech, Via Monteroni, 73100 Lecce, Italy; angelica.panico@caresilk.it; 3 Department of Engineering for Innovation, University of Salento, Via Monteroni, 73100 Lecce, Italy; annalia.masi@unisalento.it (A.M.); francesca.russo@unisalento.it (F.R.)

**Keywords:** biofilm, silver, infection, nanotechnology, antibiotic resistance

## Abstract

Background/Objectives: The huge concerns associated with biofilm-related infections in surgical procedures, along with the antibiotic resistance demonstrated by an increasing number of bacteria, have highlighted the need for alternative and effective prevention approaches. The aim of this research was to develop novel antimicrobial coatings on surgical sutures for the prevention of surgical site infections through nanotechnology-based methods. Results: The results demonstrated that although very low amounts of silver precursor were adopted for the treatments, the silver coating was effective against *Staphylococcus aureus* and antibiotic-resistant *Pseudomonas aeruginosa* in reducing the potential risk of infection. Methods: Nanostructured silver coatings were deposited onto the surface of polyglactin 910 absorbable braided sutures through a technology based on a photo-assisted chemical reaction. The materials were characterized in order to verify the efficacy of the coating in preventing biofilm formation and in reducing the bacterial colonization of the device. Conclusions: As a broad-spectrum antimicrobial agent, silver represents an important option for the prevention and management of surgical site infections. The silver deposition technology adopted in this work provides an interesting strategy for preventing biofilm formation on medical devices such as surgical sutures.

## 1. Introduction

Depending on the surgical procedure, surgical site infections (SSIs) can develop at the level of skin or subcutaneous or deep tissues and organs within 30 or 90 days of surgery. These infections represent the second highest cause of hospital-acquired infections and are a serious issue for healthcare systems in terms of morbidity, mortality and associated costs [1,2]. It has been estimated that approximately 0.5% to 3% of patients undergoing surgery are subjected to infection at the surgical incision site, with hospitalizations of 7 to 11 days longer than patients not experiencing SSIs [3], increased antibiotic use and higher treatment costs [4]. Different risk factors associated with surgery have been reported in the Centers for Disease Control and Prevention (CDC) 2018 guidelines in relation to the patient, the surgery, and pre-, intra- and postoperative processes [4] depending on the patient’s immune system, use of antibiotic prophylaxis, the degree of bacterial wound contamination and the presence of foreign material [3]. One of the most widely reported reasons for SSI development is the microbial adhesion to surgical sutures [5], which can be responsible for moving bacteria from the skin to the deeper tissues during wound closure [6]. After insertion, the surface of the suture is rapidly coated by fibrinogen, fibronectin or collagen, which can promote the adhesion of microorganisms. Moreover, the capability of bacteria to develop a matrix made of polysaccharides, proteins and nucleic acids as a protection from antimicrobial agents [6,7] represents a serious concern in the treatment of biofilm-associated SSIs due to the lack of efficacy of conventional antibiotic therapies, particularly in polymicrobial communities. Due to the multiple mechanisms of resistance developed by multidrug-resistant bacteria, biofilm-related infections, which represent approximatively 80% of SSIs, are extremely difficult to manage [7]. Some of the most common endogenous microorganisms associated with SSIs are *Staphylococcus aureus*, coagulase-negative *staphylococci*, *Enterococcus* and *Escherichia coli*, while potential exogenous sources for contamination have been found in operating rooms, air, staff members and surgical instruments [7].

On surgical sutures, the formation of biofilms can be promoted by the type of material and by the physicochemical features of the device [6,8,9], with a higher incidence associated with braided sutures than with monofilament sutures [6] due to their more complex surfaces, which facilitate the entrapment of bacteria [6,8]. In order to reduce bacterial contamination, particularly in braided sutures, the broad-spectrum antiseptic triclosan has been adopted as an effective coating against different bacteria, including antibiotic-resistant strains [5,6,10]. Although limited clinical research has been reported, other products have been recently developed, such as chlorhexidine-coated sutures [5,11] and nanosilver-coated sutures [12].

The use of nanotechnology-based approaches has transformed many aspects of clinical practice, including imaging, wound healing and surgical tools [13]. The scientific literature has reported the bactericidal effect of silver nanoparticles against Gram-positive and Gram-negative bacteria through multiple mechanisms of action, such as membrane damage, mitochondrial dysfunction, ROS generation, oxidative stress and DNA damage [14].

Silver nanoparticles have also demonstrated effectiveness in preventing bacterial biofilm formation on medical devices such as catheters and wound-dressing biomaterials, thus representing an encouraging approach for reducing the risk of SSIs associated with contaminated surgical sutures [15,16].

The aim of this research was to develop antibacterial multifilament sutures for preventing biofilm-associated infections in wound closure. Silver nanocoatings were deposited on polyglactin 910 absorbable braided sutures through a photochemical process that provided the in situ synthesis and deposition of silver particles onto the surface of the device. The silver-treated sutures were characterized by scanning electron microscopy (SEM) in order to confirm the presence of silver on the material. Most importantly, a microbiological analysis was performed to investigate the efficacy of the silver coating in preventing bacteria adhesion and biofilm formation by two representative bacteria strains responsible for SSIs, namely *Staphylococcus aureus* and the antibiotic-resistant *Pseudomonas aeruginosa*; this demonstrated the good potential of the proposed approach in terms of preventing bacterial contamination and surgical site infections.

## 2. Results

### 2.1. Morphological Characterization

**Figure 1:**
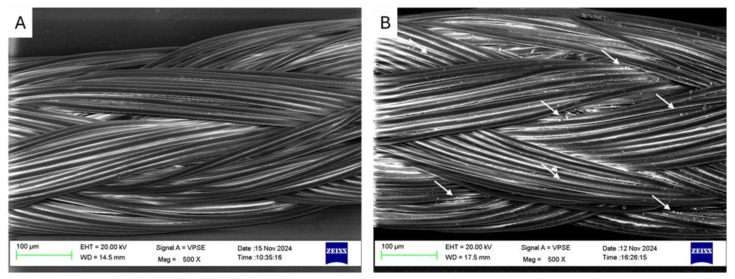
SEM images of the untreated suture (**A**) and the silver-treated sample (**B**). The arrows indicate the presence of silver particles on the surface of the material.

The efficacy of silver treatment on sutures was evaluated by scanning electron microscopy (SEM). SEM analyses evidenced the typical braided structure of these sutures (Figure 1A) and confirmed the presence of silver particles distributed on the surface of the material (Figure 1B), indicating a successful deposition treatment.

### 2.2. Microbiological Characterization

**Figure 2:**
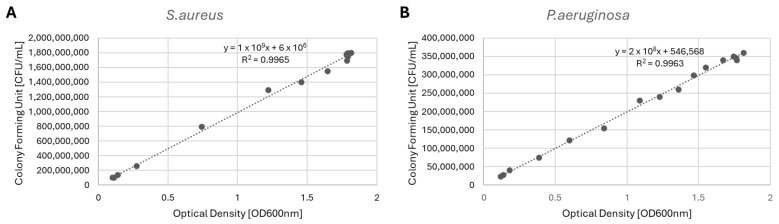
Relationship between the concentration of colony-forming units and optical density for *S. aureus* (**A**) and *P. aeruginosa* (**B**).

The growth curves of the bacteria were calculated and analyzed to define the conversion factor from OD to CFU/mL. The relation between OD and CFU/mL is reported in Figure 2. The resulting equations were used to convert the OD to CFU/mL, with 1OD = 1 × 10^9^ CFU/mL in the case of *S. aureus*, and 1OD = 2 × 10^8^ CFU/mL in the case of *P. aeruginosa*.

**Figure 3:**
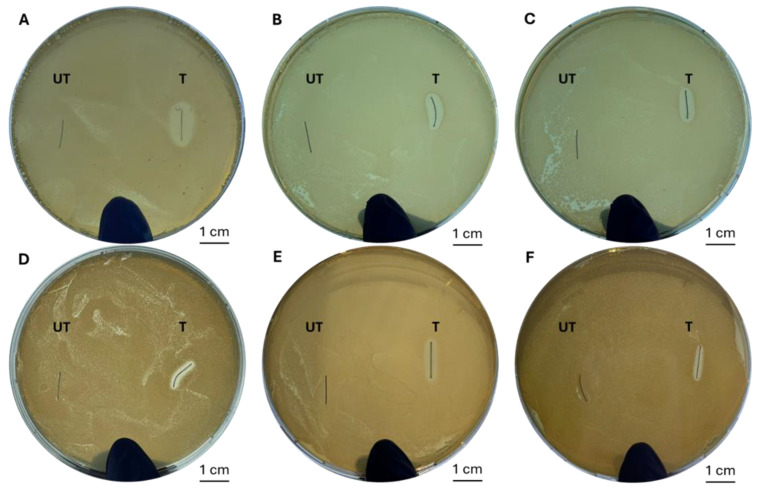
Agar diffusion tests on untreated (UT) and silver-treated (T) samples against antibiotic-resistant *Pseudomonas aeruginosa* (**A**–**C**) and *Staphylococcus aureus* (**D**–**F**) at different degradation time points, namely t = 0 (**A**,**D**), t = 1 day (**B**,**E**) and t = 3 days (**C**,**F**).

The microbiological characterization, addressed to evaluate the capability of the coating in reducing bacterial adhesion and proliferation for the prevention of biofilm formation, was performed according to different protocols. In particular, qualitative tests were performed through agar diffusion tests against the selected bacterial strains. The results reported in Figure 3 demonstrated the presence of a bacterial growth inhibition area that was larger than 1 mm in the presence of silver-treated samples (Figure 2) that, according to the Swiss Standards SN 195920:1992 [17], also indicated good antibacterial efficacy after the degradation tests. The results obtained by quantitative tests through spectrophotometric analyses are reported in Table 1, where a reduction in bacteria viability can be observed between the untreated and silver-treated samples at the initial time point and during the degradation experiment.

### 2.3. Fluorescent Imaging of Biofilms

**Figure 4:**
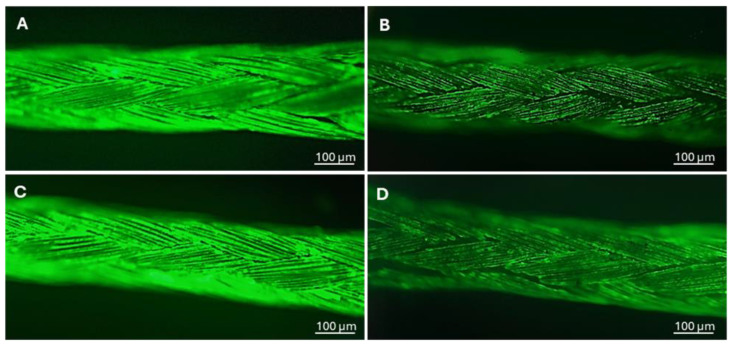
SYTO 9 images of *Pseudomonas aeruginosa* and *Staphylococcus aureus* biofilms matured respectively on the surface of untreated (**A**–**C**) and silver-treated (**B**–**D**) sutures.

A qualitative evaluation of the biofilm formation on untreated and silver-treated sutures was performed through fluorescence microscopy on *P. aeruginosa* and *S. aureus* (Figure 4). In both bacterial strains, a reduction in the adhesion of bacteria to the treated samples was observed in comparison with the untreated sutures, suggesting efficacy of the coating in inhibiting the development of the biofilm.

### 2.4. Quantification of Biofilms

**Figure 5:**
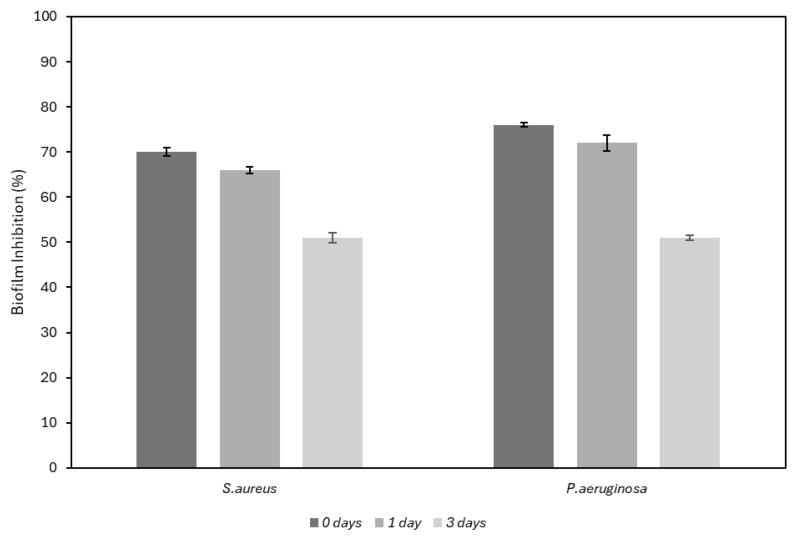
Biofilm inhibition percentage calculated in comparison with the untreated samples at t = 0, t = 1 day and t = 3 days.

A quantitative evaluation of the biofilm was also performed through the Crystal Violet assay. Figure 5 reports the percentages of biofilm inhibition calculated for both bacterial strains at different time points in comparison with the untreated samples.

### 2.5. Hemolytic Activity

Hemolytic rates of the untreated samples and the treated sutures in vitro were 1.7% and 2.1%, respectively, which, in both cases, were lower than the recommended value of 5% reported in the literature [18].

## 3. Discussion

Surgical sutures, known since 3500 BC as an integral part of surgical procedures, are manufactured today as different types, such as absorbable/non-absorbable, synthetic/natural, monofilament/braided, etc. [8]. Despite the various commercial sutures available in clinics, improving the biological performance of the currently available sutures still remains a huge challenge, particularly in terms of the prevention of wound infections [19]. Bacterial accumulation at the surgical site determines a hypoxic wound environment, inhibiting the activity of fibroblasts and resulting in delayed healing [20]. Surgical site infections (SSIs) have been recognized as a frequent complication in dermatologic surgery, with increased morbidity, impaired wound healing and increased treatment costs [21]. Every year, millions of patients are affected by SSIs worldwide, even in developed nations. In the United States, for example, this type of infection has been estimated to cause an extra 0.4 million hospital days and an added USD 10 billion in yearly expenses [22]. In Europe, the most common pathogen associated with SSIs with prolonged hospitalization and higher death rates and treatment costs is *Staphylococcus aureus* [1]; other microbial pathogens associated with SSIs include *Escherichia coli*, *Pseudomonas aeruginosa*, *Acinetobacter* species and *Enterococcus* species [22]. Drug-resistant polymicrobial biofilms often colonize suture materials, and although many antibacterial sutures are available today, their capability in preventing biofilm colonization by polymicrobial communities is still underexplored [23]. Biofilm formation on polymeric devices is a serious issue [24], and considering that antimicrobial resistance is a global threat, mutual international efforts are required to reduce the incidence of SSIs [1]. 

The complex mechanisms of biofilm formation begin by the bacterial colonization of the material, followed by the production of an extracellular substance by bacteria that provides them with protection from the outer environment and from antimicrobial agents [24]. Many efforts have been made by researchers for the development of novel, safe and efficient antibiofilm strategies as alternative methods to conventional approaches [7]; in this regard, antibiotics, salts, compounds and silver nanoparticles have been incorporated into the surface or the bulk material [24]. Moreover, depending on the type of material, different sutures have demonstrated different degrees of absorbability to bacteria, and in particular, polyglycolic acid has shown higher bacterial adhesion than monofilament polypropylene sutures [20]. The macrostructure of a braided strand is also more prone to the entrapment of bacteria and to bacterial colonization [6]. Masini et al. have evaluated the bacterial adherence to different sutures materials through a bioluminescent in vitro model focusing on poliglecaprone, polypropylene, silk, polyglycolic acid and triclosan-treated polyglycolic acid sutures. After biofilm formation, the highest bacterial count and the most statistically significant data in terms of bacterial adherence were associated with polyglycolic acid with respect to the other sutures [25]. Based on these considerations, this research work addresses the definition of antimicrobial coatings on polyglactin 910 absorbable braided sutures for the prevention of surgical site infections. As an antimicrobial agent, silver has been selected due to its well-recognized broad-spectrum antimicrobial activity, which has also been demonstrated against drug-resistant strains; in particular, at a nanometric scale [26,27]. Nanostructured silver coatings have been deposited in this work according to a technology based on the photoreduction of a silver precursor directly onto the surface of the material, followed by the conversion of the silver salt to metal silver and the in situ deposition of silver particles. This process has been successfully applied to different types of substrates for biomedical applications, and in each case, the process parameters were properly defined according to the specific application of the device by verifying the antibacterial activity, even after aging of the material and in simulated working conditions [26,27,28]. Moreover, the presented technology allows for the possibility of treating only the surface of the device rather than treating the bulk material, with important advantages in terms of costs. Indeed, optimization of the process parameters in relation to the specific application consists of defining the minimum amount of silver precursor, the proper deposition method and the minimum UV exposure time. The silver deposition technology adopted in this work and the selected process parameters provided the specific substrate with long term antibacterial properties without altering the properties of the material. Particularly, due to the specific nature of the braided sutures and their degradability, the effect of the silver treatment was investigated in terms of biofilm prevention as well as by evaluating the bacterial response to the silver coating at different degradation times to simulate the potential aging of the material. Interestingly, the antibacterial capability of the silver-treated sutures was confirmed through qualitative and quantitative assays at all time points, indicating good efficacy of the coating against both the tested bacterial strains, with similar results between *S. aureus* and *P. aeruginosa*. Moreover, a reduction in bacterial adhesion to the sutures was observed through fluorescence microscopy and the Crystal Violet assay against selected bacteria strains, including an antibiotic-resistant strain of *P. aeruginosa*. The quantification of the bacterial biofilms indicated a similar effectiveness of the coating against the tested bacterial strains, which will be further investigated in future works involving more representative microorganisms in SSIs. Moreover, the silver-treated samples did not evidence any hemolytic effects in vitro, suggesting a safe contact between the device and blood.

The activity of the silver coating in reducing bacteria proliferation and adhesion, with relevant advantages in terms of biofilm formation, suggests a potential beneficial effect of the silver-treated sutures in preventing surgical site infections.

## 4. Materials and Methods

### 4.1. Silver Treatment of Surgical Sutures

Polyglactin 910 absorbable braided sutures were selected as a substrate for the deposition of antibacterial silver coatings. The technology adopted for the silver treatment was based on a photoreduction process, which provides for the in situ synthesis and deposition of silver nanoparticles onto the surface of the material without the use of any binder. Briefly, the process consists of the deposition of a silver solution through dip coating or spray coating, followed by the exposure of the substrate to an ultraviolet (UV) source which promotes the conversion of silver salt to metal silver particles. Indeed, the silver solution was prepared by mixing silver nitrate (ACS reagent, ≥99.0%; Sigma Aldrich, St. Louis, MO, USA) as a precursor for metal silver, methanol (ACS reagent, ≥99.8%; Sigma Aldrich, St. Louis, MO, USA) as a photoreducing agent and water as solvent, and the selected percentages were 0.5 wt/wt%, 10 wt/wt% and 89.5 wt/wt%, respectively. The sutures (1 cm length) were immersed in the silver solution for 5 min and then exposed to a UV lamp (365 nm, 500 Watt; Jelosil, Milan, Italy) for 15 min. Then, silver-treated sutures as experimental samples and untreated sutures as controls were characterized at the initial time point (t = 0) and after 1 day (t = 1) and 3 days (t = 3) of degradation in 1 mL of phosphate-buffered saline (PBS).

### 4.2. Scanning Electron Microscopy

In order to evaluate the presence of the silver coating on the surface of the sutures and the distribution of the silver particles, scanning electron microscopy (SEM; Zeiss EVO, Oberkochen, Germany) was performed on the untreated and the silver-treated samples.

### 4.3. Microbiological Characterization

The microbiological characterization was performed on an antibiotic-resistant bacterial strain of *Pseudomonas aeruginosa* (ATCC BAA-3285) and *Staphylococcus aureus* (ATCC 29213). The growth curves of the bacteria were determined using the optical density (OD) method at 600 nm and colony-forming unit (CFU) calculations. Specifically, 25 mL of sterile tryptic soy broth (TSB) was inoculated with each bacterial strain to achieve an initial inoculation density of 0.1 OD. The inoculated suspensions were incubated at 37 °C in a shaking incubator (VDRL, 711/CT; ASAL, Cernusco sul Naviglio, Italy). The optical density at 600 nm (OD600) was measured using a spectrophotometer (V-1200, VWR; Radnor, PA, USA) every 1 h for 15 h to monitor bacterial growth. At each timepoint, the CFUs were calculated by the serial dilution method. Each aliquot was diluted through serial dilutions of 1:10 by transferring 100 µL of the previous dilution into the subsequent tube containing 100 µL of sterile distilled water. A volume of 100 µL of each dilution was spread onto agar plates, and after incubation, the colonies were counted to calculate the CFU/mL. The relationship between the OD and the CFU/mL was analyzed to obtain the conversion factor. The conversion factors calculated for each strain were used to convert the results of the antibacterial activity from OD to CFU/mL.

The antibacterial activity of the silver-treated sutures was determined through both qualitative and quantitative assays performed immediately after the silver treatment (t = 0) and at subsequent time points (at t = 1 day and t = 3 days) during the degradation experiments. 

The qualitative assay was performed using the agar diffusion test according to Standard “SNV 195920-1992” [17], which evaluates the antibacterial activity of the sample in relation to the width of the bacterial growth inhibition area and defines the different levels of antibacterial activity from “insufficient” (agar plate completely covered by bacteria) to “good” (inhibition zone ≥ 1 mm) [29,30]. In this experiment, 200 µL of the bacterial suspension was plated onto an agar plate, and the experimental and control samples were placed on the surface and incubated for 24 h at 37 °C (Incubator IGS100; Thermo Scientific, Waltham, MA, USA).

Quantitative tests on silver-treated and untreated suture samples were performed in duplicate using spectrophotometric analysis (Spectrophotometer V-1200, VWR), measuring the OD at 600 nm. The samples were immersed in 6 mL of tryptic soy broth inoculated with each bacterial strain at an initial OD of 0.0005 and incubated at 37 °C in a shaking incubator for 5 h (VDRL, 711/CT+; ASAL). Then, the OD at 600 nm was measured and converted into CFU/mL. The antibacterial efficiency of the samples (ABE %) was calculated using the following equation:

ABE% = ((D_c_ − D_t_)/D_c_) × 100 (1)

where D_c_ and D_t_ are the CFU/mL values in the presence of the untreated and treated sample respectively [31].

### 4.4. Fluorescent Imaging of Biofilms

In order to evaluate the effect of the silver coating on biofilm formation, the samples were immersed in 6 mL of tryptic soy broth inoculated with each bacterial strain at an initial OD = 0.0005 and incubated at 37 °C under shaking conditions for 48 h. After incubation, the samples were washed with phosphate-buffered saline (PBS) to remove non-adherent bacterial cells and then dried at 37 °C for 1 h. *Pseudomonas aeruginosa* and *Staphylococcus aureus* biofilms matured on the surface of silver-treated and untreated sutures were observed using fluorescence microscopy. The biofilms were stained using a green fluorescent nucleic acid stain at a concentration of 3 µL (SYTO9; Molecular Probes, Waltham, MA, USA). After incubation for 15 min in the dark, the samples were washed with PBS, dried at 37 °C and observed with a fluorescence microscope at 10× (Axio Vert A1 Zeiss).

### 4.5. Quantification of Biofilms

As in previous characterizations, after immersion in 6 ml of tryptic soy broth inoculated with each bacterial strain (OD = 0.0005) and incubation at 37 °C under shaking conditions for 48 h, the samples were washed in PBS to remove non-adherent bacterial cells and dried at 37 °C for 1 h. Then, to quantify the antibiofilm activity of the silver coatings, the Crystal Violet (Sigma Aldrich) assay was performed. The samples were stained at room temperature with a 0.1% solution of Crystal Violet for 15 min. Then, the samples were washed with PBS and immersed in 95% ethanol for 20 min. The concentration of Crystal Violet was determined using a spectrophotometer by measuring the optical density at 595 nm (V-1200, VWR). The experiment was performed in duplicate. The percentage of biofilm inhibition was calculated as in Equation (1).

### 4.6. Hemolytic Activity

The hemolysis test was performed using an indirect contact method to evaluate the hemocompatibility of silver-treated suture threads. The silver-treated and untreated samples were immersed in a 0.9% saline solution for 24 h to obtain the extraction solution. 10 mL of sheep blood was centrifugated at 3000 rpm for 10 min to separate the red blood cells from the plasma. The cell pellet was washed three times and resuspended in 10 mL of saline solution. A 500 µL aliquot of the extraction solution and 500 µL of the blood cell suspension were incubated for 1 h at 37 °C in a thermoblock (TD 150 P1; Falc Instruments, Treviglio, Italy). The negative control group included saline solution and blood without the sample, while the positive control group consisted of distilled water. After centrifugation at 15,000 rpm for 15 min (Laboratory Microcentrifuge D3024; Scilogex, Rocky Hill, CT, USA), the supernatant was collected for absorbance (A) measurement at 540 nm. The hemolysis rate was calculated using the following equation [18]:

Hemolytic rate = (A_material_ − A_negative control_)/(A_positive control_ − A_negative control_) × 100 (2)

## 5. Conclusions

Biofilm-related surgical site infections represent a serious issue for healthcare systems in terms of morbidity, mortality and costs. Some devices such as surgical sutures, in particular, can become colonized by bacteria to varying extents depending on the specific surgical procedure and the structure and the material of the device, thus contributing to an increased risk of infections. In addition, the resistance to antibiotics demonstrated by many bacteria has limited the number of therapeutic options. In this scenario, many efforts have been made by the scientific research community to define novel and effective strategies for preventing bacterial proliferation. Silver, a well-known broad-spectrum antimicrobial agent, has been used in this work to obtain surgical sutures with antimicrobial properties. Nanostructured silver coatings were developed and characterized against representative bacterial strains in SSIs. The results demonstrated effectiveness in reducing bacterial proliferation and biofilm formation on surgical sutures and could be considered an interesting option against surgical site infections.

## Figures and Tables

**Table 1:** Quantification of the antibacterial efficacy (ABE; %).

**Bacteria**	**Degradation** **Days**	**Materials**	**CFU/mL After 5** **h**	**ABE (%)**
*S. aureus*	0 days	Untreated Ag/suture	2.37 × 10^8^ 2.90 × 10^7^	- 88
	1 days	Untreated Ag/suture	2.41 × 10^8^ 3.50 × 10^7^	- 85
	3 days	Untreated Ag/suture	2.45 × 10^8^ 4.25 × 10^7^	- 83
*P. aeruginosa*	0 days	Untreated Ag/suture	3.50 × 10^7^ 2.25 × 10^6^	- 94
	1 days	Untreated Ag/suture	3.49 × 10^7^ 3.45 × 10^6^	- 90
	3 days	Untreated Ag/suture	3.34 × 10^7^ 4.05 × 10^6^	- 88

## Data Availability

Data are contained within the article.

## References

[1] Mellinghoff S.C., Bruns C., Albertsmeier M., Ankert J., Bernard L., Budin S., Bataille C., Classen A.Y., Cornely F.B., Couvé-Deacon E. (2023). Staphylococcus Aureus Surgical Site Infection Rates in 5 European Countries. Antimicrob. Resist. Infect. Control.

[2] Liston J., Bayles A. (2023). Surgical Site Infections. Surgery.

[3] Seidelman J.L., Mantyh C.R., Anderson D.J. (2023). Surgical Site Infection Prevention. JAMA.

[4] Maraş G., Sürme Y. (2023). Surgical Site Infections: Prevalence, Economic Burden, and New Preventive Recommendations. Explor. Res. Hypothesis Med..

[5] Yang Y., Zhou Z., Ma R., Ren J., Wu X. (2024). Antimicrobial-Coated Sutures versus Non-Coated Sutures in Reducing Surgical Site Infection: An Updated Systematic Review and Meta-Analysis. J. Hosp. Infect..

[6] Dhom J., Bloes D.A., Peschel A., Hofmann U.K. (2017). Bacterial Adhesion to Suture Material in a Contaminated Wound Model: Comparison of Monofilament, Braided, and Barbed Sutures. J. Orthop. Res..

[7] Hrynyshyn A., Simões M., Borges A. (2022). Biofilms in Surgical Site Infections: Recent Advances and Novel Prevention and Eradication Strategies. Antibiotics.

[8] Li H., Wang Z., Robledo-Lara J.A., He J., Huang Y., Cheng F. (2021). Antimicrobial Surgical Sutures: Fabrication and Application of Infection Prevention and Wound Healing. Fibers Polym..

[9] Asher R., Chacartchi T., Tandlich M., Shapira L., Polak D. (2019). Microbial Accumulation on Different Suture Materials Following Oral Surgery: A Randomized Controlled Study. Clin. Oral. Investig..

[10] He P., Liu Z., Chen H., Huang G., Mao W., Li A. (2023). The Role of Triclosan-Coated Suture in Preventing Surgical Infection: A Meta-Analysis. Jt. Dis. Relat. Surg..

[11] Carella S., Fioramonti P., Onesti M.G., Scuderi N. (2019). Comparison between Antimicrobial-Coated Sutures and Uncoated Sutures for the Prevention of Surgical Site Infections in Plastic Surgery: A Double Blind Control Trial. Eur. Rev. Med. Pharmacol. Sci..

[12] Mathew S., Vijaya Kumar K., Prabhu A., Shastry R.P., Rajesh K.S. (2024). Braided Silk Sutures Coated with Photoreduced Silver Nanoparticles for Eradicating Staphylococcus Aureus and Streptococcus Mutans Infections. J. Microbiol. Methods.

[13] Abaszadeh F., Ashoub M.H., Khajouie G., Amiri M. (2023). Nanotechnology Development in Surgical Applications: Recent Trends and Developments. Eur. J. Med. Res..

[14] Liao C., Li Y., Tjong S.C. (2019). Bactericidal and Cytotoxic Properties of Silver Nanoparticles. Int. J. Mol. Sci..

[15] Cooper I.R., Pollini M., Paladini F. (2016). The Potential of Photo-Deposited Silver Coatings on Foley Catheters to Prevent Urinary Tract Infections. Mater. Sci. Eng. C.

[16] Pollini M., Paladini F., Licciulli A., Maffezzoli A., Sannino A. (2012). Engineering Nanostructured Silver Coatings for Antimicrobial Applications. Nano-Antimicrobials.

[17] (1992). Textile Fabrics—Determination of the Antibacterial Activity—Agar Diffusion Plate Test.

[18] Luna-Vázquez-Gómez R., Arellano-García M.E., García-Ramos J.C., Radilla-Chávez P., Salas-Vargas D.S., Casillas-Figueroa F., Ruiz-Ruiz B., Bogdanchikova N., Pestryakov A. (2021). Hemolysis of Human Erythrocytes by Argovit^TM^ AgNPs from Healthy and Diabetic Donors: An In Vitro Study. Materials.

[19] Li Y., Meng Q., Chen S., Ling P., Kuss M.A., Duan B., Wu S. (2023). Advances, Challenges, and Prospects for Surgical Suture Materials. Acta Biomater..

[20] Mahesh L., Kumar V., Jain A., Shukla S., Aragoneses J., Martínez González J., Fernández-Domínguez M., Calvo-Guirado J. (2019). Bacterial Adherence Around Sutures of Different Material at Grafted Site: A Microbiological Analysis. Materials.

[21] Schlager J.G., Patzer K., Wallmichrath J., French L.E., Kunrad E., Schlingmann S., Stiefel D., Kendziora B., Hartmann D. (2023). Surgical Site Infection in Skin Surgery—An Observational Study. Int. Wound J..

[22] Suleiman A.S., Abbass M., Hossain M., Choudhary P., Bhattacharya P., Islam M.A. (2023). Impact of Antibiotic-Coated Sutures on Surgical Site Infections: A Second-Order Meta-Analysis. Int. J. Surg..

[23] Prabha S., Sowndarya J., Ram P.J.V.S., Rubini D., Hari B.N.V., Aruni W., Nithyanand P. (2021). Chitosan-Coated Surgical Sutures Prevent Adherence and Biofilms of Mixed Microbial Communities. Curr. Microbiol..

[24] Kaali P., Strmberg E., Karlsso S. (2011). Prevention of Biofilm Associated Infections and Degradation of Polymeric Materials Used in Biomedical Applications. Biomedical Engineering, Trends in Materials Science.

[25] Masini B.D., Stinner D.J., Waterman S.M., Wenke J.C. (2011). Bacterial Adherence to Suture Materials. J. Surg. Educ..

[26] Sportelli M.C., Picca R.A., Paladini F., Mangone A., Giannossa L.C., Di Franco C., Gallo A.L., Valentini A., Sannino A., Pollini M. (2017). Spectroscopic Characterization and Nanosafety of Ag-Modified Antibacterial Leather and Leatherette. Nanomaterials.

[27] Picca R.A., Paladini F., Sportelli M.C., Pollini M., Giannossa L.C., Di Franco C., Panico A., Mangone A., Valentini A., Cioffi N. (2017). Combined Approach for the Development of Efficient and Safe Nanoantimicrobials: The Case of Nanosilver-Modified Polyurethane Foams. ACS Biomater. Sci. Eng..

[28] Paladini F., Pollini M. (2022). Novel Approaches and Biomaterials for Bone Tissue Engineering: A Focus on Silk Fibroin. Materials.

[29] Pantulap U., Unalan I., Zheng K., Boccaccini A.R. (2024). Hydroxycarbonate Apatite Formation, Cytotoxicity, and Antibacterial Properties of Rubidium-Doped Mesoporous Bioactive Glass Nanoparticles. J. Porous Mater..

[30] Miola M., Bruno M., Maina G., Fucale G., Lucchetta G., Vernè E. (2014). Antibiotic-Free Composite Bone Cements with Antibacterial and Bioactive Properties. A Preliminary Study. Mater. Sci. Eng. C.

[31] Mukherjee S., Chowdhury D., Kotcherlakota R., Patra S., Vinothkumar B., Bhadra M.P., Sreedhar B., Patra C.R. (2014). Potential Theranostics Application of Bio-Synthesized Silver Nanoparticles (4-in-1 System). Theranostics.

